# Spheroidization of Nickel Powder and Coating with Carbon Layer through Laser Heating

**DOI:** 10.3390/ma11091641

**Published:** 2018-09-07

**Authors:** Shuang Li, Yu-Ling Shao, Lan Cui, Sergei A. Kulinich, Xi-Wen Du

**Affiliations:** 1 School of Materials Science and Engineering, Tianjin University, Tianjin 300072, China; 15632301425@163.com (S.L.); yulingshao@163.com (Y.-L.S.); cuilan@tju.edu.cn (L.C.); 2 Research Institute of Science & Technology, Tokai University, Kanagawa 259-1292, Japan; skulinich@tokai-u.jp

**Keywords:** nickel powder, carbon-encapsulated, long-pulse-width laser, oxidation resistance

## Abstract

We developed a simple and efficient process, laser heating of nickel powder in ethanol, to produce carbon-encapsulated nickel microspheres. Long-pulse-width laser heated nickel powder suspended in pure ethanol into liquid droplets. In turn, the latter droplets became sphere-like, pyrolyzed surrounding ethanol and dissolved the produced carbon atoms. Because of their lower solubility in solid nickel, excess carbon atoms were then expelled from the metal core after solidification, thus forming graphite-like shells on the laser-modified Ni spheres. Hence, after pyrolysis the transformation of carbon was found to follow the dissolution-precipitation mechanism. The produced carbon-encapsulated nickel microspheres exhibited higher oxidation resistance compared with the initial nickel powder, while keeping their magnetic properties essentially unchanged.

## 1. Introduction

Powders of active metals have been actively applied in many fields, being used as catalysts, conductive coatings and electrodes, to name just a few. However, when exposed to high temperature and/or humid environment, they are known to show poor oxidation resistance [1,2,3,4]. Extensive investigations have been made to improve the resistance of such nanopowders without compromising their functionalities, the most efficient strategies found being the introduction of additives (e.g., Ag [1,2], Cu [3], and ZrO_2_ [4]) or the formation of a protective carbon layer [5,6,7,8,9,10,11,12,13,14]. So far, many techniques have been developed to coat a carbon layer over metal nanoparticles, such as arc discharge [5,6], magnetron sputtering [7], hydrothermal carbonization [8], detonation [9], chemical vapor deposition [10,11], spray pyrolysis [12], and pyrolysis of organometallic compounds [13,14]. Despite the noticeable success achieved, all the above methods either require severe experimental conditions or suffer low purity. Therefore, new efficient approaches for preparing carbon-encapsulated metal particles (CEMPs) are still anticipated.

Laser ablation of a metal target in liquid media has been regarded as an efficient and green way for coating carbon layers onto particles of various metals. Duan et al. reported on preparation of Au@C nanostructures via laser ablation of a gold target in mixed solutions of toluene and ethanol [15]. Amendola and coworkers fabricated magnetic Fe@C nanoparticles (NPs) by ablating bulk iron in various organic solvents [16]. Core-shell Fe@C NPs were laser-produced by Yu et al. from iron in methanol-dissolved ascorbic acid [17]. Carbon-encapsulated nickel NPs and carbon-encapsulated platinum NPs were reported in work [18] to be more stable and efficient in dye-sensitized solar cells. Core-shell Pd@C NPs were produced by laser ablation of Pd foil submerged in acetonitrile, demonstrating higher catalytic efficiency as catalyst for nitrobenzene-to-aniline reduction compared with uncoated Pd NPs [19]. Typically, fast lasers with pulse widths of several nanoseconds or picoseconds are employed to ablate the metal target and generate plasma (vapor, or metal droplets), which further condenses into NPs. Simultaneously, depending on liquid, a carbon layer may form on the surface of metal core through pyrolysis of organic medium [20,21,22]. Although laser ablation has been proved successful in the production of nano-sized CEMPs, so far it has never been utilized to coat carbon layer onto metallic particles with micron sizes.

Unlike their fast and ultrafast counterparts, long-pulse-width lasers, i.e., millisecond pulsed lasers, possess rather low power densities (e.g., on the order of 100 W/cm^2^) [23,24,25,26,27,28], which typically causes linear absorption [29] and Joule heating [30] of the target. Therefore, the temperature rises of micropowder particles subjected to long-pulse-width-laser irradiation can be controlled by tuning laser parameters. Correspondingly, at appropriate temperatures, molten metal particles are expected to form and decompose the organic liquid used as medium. During quenching, the generated carbon atoms should form a shell around cooling particles, resulting in CEMPs. As a “proof of concept”, this study aimed to demonstrate that carbon-encapsulated nickel particles (CENPs) could be produced by laser irradiation of nickel powder suspended in anhydrous ethanol. The prepared CENPs were found to exhibit higher oxidation resistance than the precursor (pure) nickel powder, and the generated carbon layer is shown not to deteriorate the performance of the Ni powder. 

## 2. Materials and Methods 

CENPs were synthesized by laser irradiation of a nickel powder in anhydrous ethanol. The Ni powder with a purity of 99.99% was purchased from Shanghai TE Connectivity. The powder was dispersed in anhydrous ethanol and irradiated by an Nd: YAG laser (wavelength 1064 nm, pulse duration from 0.1 to 20 ms, frequency from 1 to 20 Hz, spot size 7 mm, single-pulse energy from 1.19 to 32.3 J/pulse). All the experiments were performed at ambient temperature and normal pressure. The liquid was placed in the beaker and was constantly sonicated to keep the powder well dispersed during laser irradiation. The samples were irradiated vertically by the laser, the irradiation time varying from one-pulse to 30 min. The as-prepared powder was molded, grinded and polished to obtain a cross sectional sample for elemental line scanning analysis.

The morphology and composition of the product were characterized by scanning electron microscopy (SEM, Hitachi S-4800, Tokyo, Japan) equipped with energy-dispersive X-ray spectroscopy (EDS) module, and by transmission electron microscopy (TEM, FEI Technai G2 F20 tool equipped with a field emission gun, Hillsboro, OR, USA). Thermogravimetric analysis (TGA) was carried out in a Pyris TGA7 thermogravimeter (Perkin-Elmer Corporation, Waltham, MA, USA). For the measurement of magnetic properties, samples were washed with deionized water for five times, dried at 20 °C for 24 h, and then analyzed in the PPMS-6000 system (from Quantum Design, San Diego, CA, USA).

## 3. Results and Discussion

**Figure 1:**
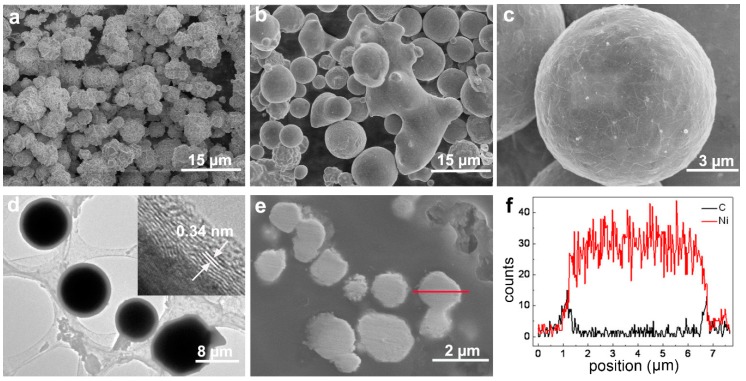
Morphology and composition of Ni particles before and after laser irradiation. (**a**) SEM image of raw (precursor) Ni particles. (**b**) SEM image of Ni particles irradiated for 30 min at laser energy of 20.2 J/pulse. (**c**) High magnification SEM image of a single particle in panel. (**d**) TEM image of laser-treated Ni particles with carbon coating, the inset is a HRTEM image showing a surface carbon layer. (**e**) SEM image of cross-sectional sample with laser-treated Ni particles. (**f**) Elemental line-scan profiles along the red line shown in (**e**).

The precursor Ni particles exhibited angular and irregular shapes with sizes varying from several hundred nanometers to 10 microns (Figure 1a). After 30 min of laser irradiation (with pulse energy of 20.2 J/pulse), the particles were found to display a spherical shape, some of them being fused together (Figure 1b). The high-magnification SEM image shown in Figure 1c illustrates that a wrinkled layer formed on the microsphere surface, supporting surface modification caused by laser irradiation. The TEM image in Figure 1d shows a core-shell structure with the shell thickness of several hundred nanometers. The high resolution TEM (HRTEM) image shown in the inset of Figure 1d reveals a thin (several nm in thickness) outer layer covering particles and demonstrating a characteristic interplanar spacing, 0.34 nm, of graphite (001) planes. Elemental line-scan analysis was performed across the particle shown in Figure 1e. The results are presented in Figure 1f, clearly indicating the particle is metallic Ni with a transition layer with carbon and nickel on its surface, the latter layer being up to several hundred nm thick, as well seen in Figure 1e.

**Figure 2:**
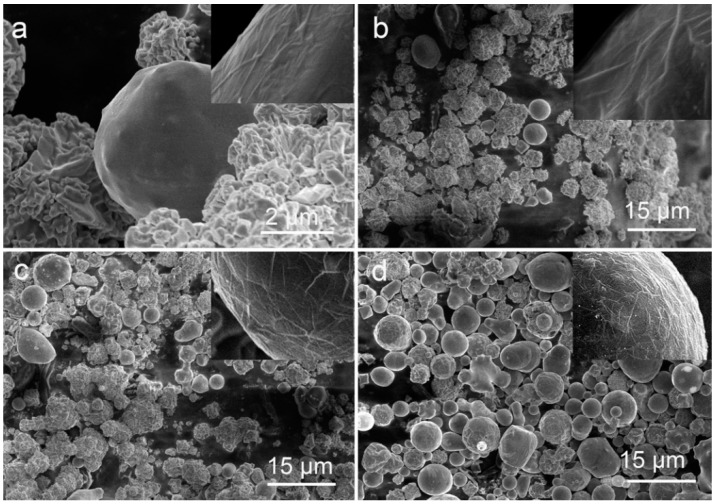
SEM images of Ni powder samples irradiated for different times at laser energy of 20.2 J/pulse. (**a**) One pulse, (**b**) 2 min, (**c**) 10 min, (**d**) 30 min. The inserts in panels (**a**–**d**) show high magnification SEM images of carbon layer on the particles.

Figure 2 displays the morphology changes observed over time as the precursor powder was irradiated with the laser energy of 20.2 J/pulse. According to SEM image in Figure 2a, the nickel particles could be molten just after one laser pulse, although the number of such molten particles was not large. More and more irregular particles were found to be molten and transformed into spherical ones as the irradiation time was prolonged to 2, 10, and then 30 min (Figure 2b–d). At the same time, a wrinkled carbon layer was always found to cover smooth spherical particles (see insets of Figure 2a–d).

**Figure 3:**
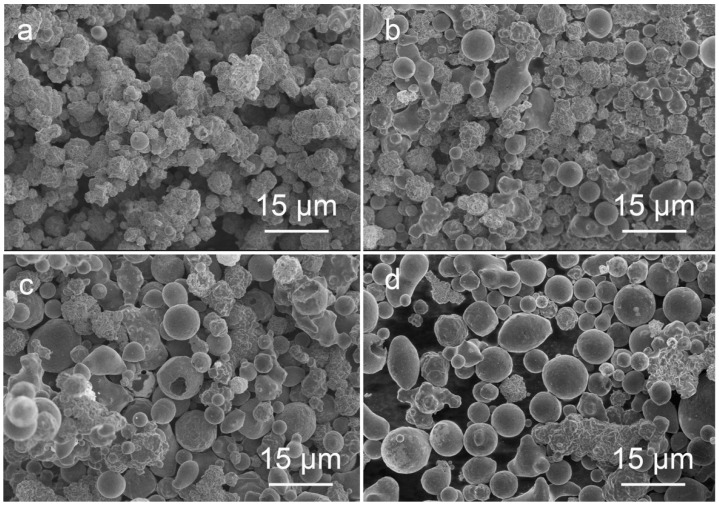
SEM images of samples irradiated for 20 min at different laser energy densities: (**a**) 21.0 J/cm^2^, (**b**) 52.4 J/cm^2^, (**c**) 72.3 J/cm^2^, and (**d**) 83.9 J/cm^2^.

Laser energy density was also found to play an important role in the formation of carbon encapsulated nickel spheres. As seen in Figure 3a, the initial nickel powder hardly transformed into core-shell spheres at a low laser fluence of 21.0 J/cm^2^, as even after 20 min of irradiation only a few smaller spheres with sizes below 4 μm can be observed in panel (a). As the laser energy density was elevated to 52.4 J/cm^2^, more sphere-shaped particles with sizes ~8 μm emerged in the product, while those larger than 10 μm exhibit partially molten features and irregular shapes (Figure 3b). Further increase in laser fluence is well seen in Figure 3c,d to result in the formation of bigger spheres, with sizes larger than 10 μm, with their fraction in the product being remarkably enhanced. 

**Figure 4:**
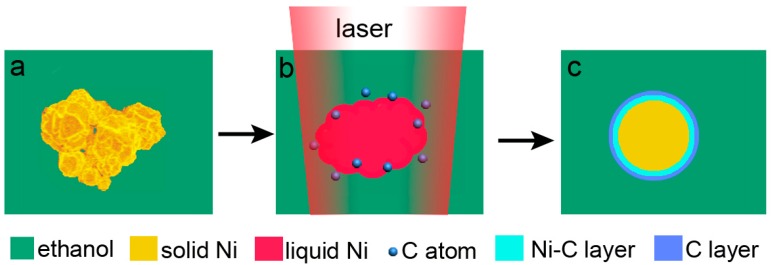
Schematic illustration of the formation of carbon-encapsulated nickel particles occurring under laser beam irradiation. (**a**) Initial particle; (**b**) molten Ni particle surrounded by pyrolyzed ethanol under laser irradiation; (**c**) final CENP.

On the basis of the above described results, the following mechanism of CENP formation is proposed. The transformation from irregular nickel particles to well-shaped spheres suggests that, when irradiated by long-pulse-width laser beam, the powder was molten via the Joule heating (Figure 4b). The molten metallic-nickel particles had to have a temperature at least as high as 1455 °C, which is high enough to pyrolyze surrounding ethanol and generate free carbon atoms (Figure 4b). On the other hand, liquid nickel is known to dissolve more than 10% of carbon atoms, whereas their solubility in solid nickel decreases to less than 2.7% after solidification [31]. Therefore, formation of a carbon-based layer is expected around each molten particle during its cooling, thus leading to a graphite-like layer well-observed in Figure 1d. A transition layer based on carbon-enriched nickel is also expected as a result of a strong quenching effect from liquid medium (Figure 4c). Similar mechanisms were previously reported by others for carbon-enriched iron- and nickel-materials, which also followed melting, organic solvent decomposition, carbon dissolution, and precipitation on metal surface [32,33].

**Figure 5:**
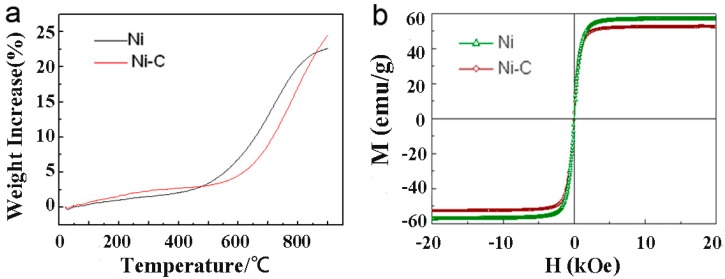
Physical properties of initial Ni powder and carbon-encapsulated nickel particles (CENPs). (**a**) TGA curves measured in air and (**b**) M–H curves tested at room temperature.

Figure 5 compares physical properties of the produced CENPs with those of as-supplied initial material. As shown in panel (a), when heated in air, the raw nickel powder shows an obvious weight increase at 425 °C, while the laser-produced CENPs remain stable up to 550 °C. This indicates that the CENPs are more stable in oxygen-containing atmosphere when compared with their nickel precursor powder whose surface was not protected. At the same time, as well seen in Figure 5b, both the as-supplied nickel powder and laser-modified CENPs exhibit paramagnetic behavior at room temperature. The presence of the carbon layer is seen somewhat to weaken the saturation magnetization of the new particles. This is believed to be a result of a “shield effect” from the dense non-magnetic graphite-like layer covering their surface which is well seen in Figure 1d. However, the observed drop in saturation magnetization of the newly prepared CENPs is not significant, being on the order of ~3% (compare the values 57 and 59 emu/g in Figure 5b). Keeping in mind the gain in oxidation resistance reached by the CENPs in comparison with the precursor Ni powder (see Figure 5a), this drop in magnetic properties is relatively small, making us believe that the carbon-based layer formed during laser treatment does not compromise the physical properties of the metallic core much, while protecting it from deterioration caused by surface oxidation. 

## 4. Conclusions

In summary, carbon-encapsulated nickel microspheres were prepared via irradiating nickel powder with millisecond pulsed laser in ethanol. The transformation was found to follow a dissolution-precipitation mechanism, where laser irradiation first heats metallic particles and generates hot droplets, after which surrounding ethanol is pyrolyzed and gives rise to carbon atoms that dissolve in liquid nickel. After solidification, because of lower solubility in solid nickel, such excessive carbon atoms are expelled from the metallic core and form a graphite-like layer on the laser-modified Ni spheres. The oxidation-resistance of as-prepared carbon-encapsulated nickel spheres was found to be improved, whereas their magnetic property did not deteriorate significantly. The demonstrated one-step technique is facile and effective, and as such it is expected to be widely applied for treatment of various metallic powders to improve their resistance to oxidation through carbon coating.
